# *Staphylococcus aureus* sequence type (ST) 45, ST30, and ST15 in the gut microbiota of healthy infants — persistence and population counts in relation to ST and virulence gene carriage

**DOI:** 10.1007/s10096-022-04539-9

**Published:** 2023-01-23

**Authors:** Forough L. Nowrouzian, Liselott Svensson Stadler, Anna Östblom, Erika Lindberg, Gerard Lina, Ingegerd Adlerberth, Agnes E. Wold

**Affiliations:** 1 Department of Infectious Diseases, Institute of Biomedicine, University of Gothenburg, Guldhedsgatan 10, S-413 46, Gothenburg, Sweden; 2 Culture Collection University of Gothenburg, Sahlgrenska Academy, University of Gothenburg, Gothenburg, Sweden; 3 Department of Clinical Microbiology, Dahlgren’s University Hospital, Region Västra Götaland, Gothenburg, Sweden; 4 Centre National de Référence Des Staphylocoques, Hospices Civils de Lyon, CIRI, Université Lyon1, INSERM U1111, CNRS-UMR 5308, Ecole Normale Supérieure de Lyon, Lyon, France

**Keywords:** *Staphylococcus aureus*, Intestinal colonization, Sequence type (ST), Virulence gene, Adhesins, Superantigen, Persistence, Population counts

## Abstract

*Staphylococcus aureus* colonizes the anterior nares, and also the gut, particularly in infants. *S. aureus* is divided into lineages, termed clonal complexes (CCs), which comprise closely related sequence types (STs). While CC30 and CC45 predominate among nasal commensals, their prevalence among gut-colonizing *S. aureus* is unknown. Here, 67 gut commensal *S. aureus* strains from 49 healthy Swedish infants (aged 3 days to 12 months) were subjected to multi-locus sequence typing. The STs of these strains were related to their virulence gene profiles, time of persistence in the microbiota, and fecal population counts. Three STs predominated: ST45 (22% of the strains); ST15 (21%); and ST30 (18%). In a logistic regression, ST45 strains showed higher fecal population counts than the others, independent of virulence gene carriage. The lower fecal counts of ST15 were linked to the carriage of *fib* genes (encoding fibrinogen-binding proteins), while those of ST30 were linked to *fib* and *sea* (enterotoxin A) carriage. While only 11% of the ST15 and ST30 strains were acquired after 2 months of age, this was true of 53% of the ST45 strains (*p* = 0.008), indicating that the former may be less fit for establishment in a more mature microbiota. None of the ST45 strains was transient (persisting < 3 weeks), and persistent ST45 strains colonized for significantly longer periods than persistent strains of other STs (mean, 34 vs 22 weeks, *p* = 0.04). Our results suggest that ST45 strains are well-adapted for commensal gut colonization in infants, reflecting yet-unidentified traits of these strains.

**Supplementary Information:**

The online version contains supplementary material available at 10.1007/s10096-022-04539-9.

## Introduction

While *Staphylococcus aureus* is one of the most-common bacterial pathogens [1], it is also a colonizer of the skin, particularly in the anterior nares [2]. More recently, it has become recognized as an important member of the commensal gut microbiota of infants [3, 4]. In fact, *S. aureus* is more commonly found in fecal samples than in nasal samples of infants and young children [5], and the strains persist for longer periods of time in the gut than in the nostrils [5]. During the first 6 months of life, 50–60% of infants have *S. aureus* in the gut microbiota [4, 5]. After 6 months of age, fecal populations of *S. aureus* decrease and the colonization rate declines [4], such that only one-quarter of adults have *S. aureus* in their feces, and often in low numbers [6]. It seems likely that the poorly developed microbiota of infants and young children exert only limited resistance to *S. aureus* colonization, and that the gut microbiota acts as an important reservoir for *S. aureus* strains that cause infections, in both infants and young children [3, 7, 8].

*S. aureus* adhesins and toxins may serve as both virulence factors and enable commensalism. Thus, fibronectin-binding proteins are associated with the capacity to colonize the gut and with long-term persistence both in the nose and in the gut [5]. Furthermore, strains that carry the adhesin gene *cna*, which encodes the collagen binding-protein, and genes that encode the superantigen genes M and O have higher fecal population levels than strains that lack these traits [9]. In contrast, strains that carry the genes for *S. aureus* enterotoxin A (*sea*) exhibit lower than average population counts in the feces [9].

The population structure of *S. aureus* is highly clonal and comprises lineages that are termed “clonal complexes” (CCs), which differ in terms of their genomic makeup. These CCs can be identified by multi-locus sequence typing (MLST), which detects polymorphisms in several housekeeping genes. One CC is comprised of closely related sequence types (STs), which differ from the founder ST by a single allele [10]. Five CCs dominate the methicillin-susceptible and methicillin-resistant *S. aureus* collections, namely CC5, 8, 22, 30, and 45 [10–15]. Globally, CC30 and CC45 appear to be most common [10, 11, 16, 17], CC30 being more prevalent among invasive strains [18], and CC45 isolates more prevalent among nasal commensal isolates [18, 19]. There is a strong linkage between CC and the variety of *agr* alleles in the *agr* operon, which encodes a quorum-sensing system that regulates a number of virulence genes [20]. Thus, ST15 strains (CC15) have *agr* II, ST30 strains (CC30) have *agr* III, and ST45 strains (CC45) have *agr* I [14, 20]. Furthermore, ST30 (CC30) strains often carry the superantigen-encoding genes *sea* and *tst* and the adhesin-encoding genes *bsp* and *cna* [14].

Uncovering the population structures of *S. aureus* commensal and invasive strains would expand our knowledge of bacterial commensalism and pathogenicity and how these phenomena interact with each other. However, to the best of our knowledge, the clonal structure of gut commensal *S. aureus* strains has been little studied. Here, we investigated the clonal structure of *S. aureus* strains derived from the gut microbiota of healthy infants who were followed longitudinally from 3 days to 12 months of age with regular quantitative culturing of fecal samples. For each strain, the sequence type was related to the virulence profile, the age at which it first appeared in the gut microbiota, fecal population counts, and its capacity to persist in the microbiota.

## Materials and methods

### *S. aureus* strain collection

The 67 *S. aureus* strains examined here were previously isolated from fecal samples obtained from 49 Swedish full-term healthy infants born in Gothenburg, Sweden, in the period 1998–1999. The infants participated in the ALLERGYFLORA birth-cohort study, which has investigated the relationship between the gut microbiota and allergy development [21].

*S. aureus* was isolated from rectal swabs obtained at 3 days of age and from fecal samples obtained at 1, 2, 4, and 8 weeks and at 6 and 12 months of age and cultured quantitatively using a previously described methodology [4, 21]. As part of a previous study, the individual *S. aureus* strains were identified by the random amplified polymorphic DNA (RAPD) method and categorized as persistent (present for ≥ 3 weeks in the gut microbiota) or transient (present for < 3 weeks) [4]. This way to categorize gut colonizing strains has been used since the 1950s [22] and enables the identification of clones or bacterial characteristics associated with long-term colonization. Notably, some strains could not be categorized as either persistent or transient; these were strains that were detected on only a single sampling occasion at 2, 6, or 12 months of age, such that the sampling intervals were too long to ensure that the strain had colonized for a period shorter than 3 weeks [4].

Each strain was previously screened for the four main allelic variants of the accessory gene regulatory (*agr*) locus and for carriage of 30 virulence genes using multiplex PCR [9]. Virulence genes included those that encoded (a) microbial surface adhesins, including fibrinogen-binding protein (*fib*), clumping factors A and B (*clfA* and *clfB*), elastin-binding protein (*ebp*), laminin-binding protein (*lbp*), collagen-binding protein (*cna*), bone sialoprotein-binding protein (*bsp*), and fibronectin-binding proteins A and B (*fnbA* and *fnbB*), and (b) *S. aureus* exotoxins, some with superantigenic properties, such as the classical *S. aureus* enterotoxins (SEA, SEB, etc.), enterotoxin gene cluster (*egc*) superantigens SElM and SElO, and toxic shock syndrome toxin TSST-1, as well as some exotoxins without superantigenic activities, such as β-hemolysin [9].

### Identification of *S. aureus* STs/CCs

The sequence types of *S. aureus* strains were determined using MLST. *S. aureus* strains that were stored frozen were cultivated aerobically on horse blood agar plates at 37 °C overnight and subcultured to ensure purity. For the preparation of bacterial DNA, a small amount of bacteria was picked from a single colony and suspended in 50 μL of 1 × Tris–EDTA buffer (Sigma Chemical Co., St. Louis, MO, USA). The mixture was incubated for 10 min at 95 °C, centrifuged at 13,400 rpm for 5 min, and the DNA-containing supernatant was collected and stored at 4 °C until analyzed. MLST was performed according to pubMLST (https://pubmlst.org/) by sequencing internal fragments of the following seven housekeeping genes: *arcC* (carbamate kinase); *aroE* (shikimate dehydrogenase); *glpF* (glycerol kinase); *gmk* (guanylate kinase); *pta* (phosphate acetyltransferase); *tpi* (triosephosphate isomerase); and *yqiL* (acetyl coenzyme A acetyltransferase), using the appropriate forward and reverse primers (https://pubmlst.org/). The assembled sequences were cut to the correct length and the corresponding STs were obtained from the pubMLST webpage (https://pubmlst.org/).

To define the relationships between the different ST types, the eBURSTv3 (Based Upon Related Sequence Types; http://eburst.mlst.net/) software was used. Clonal complexes were defined as closely related STs that shared at least five of seven alleles with one other ST in the group and also shared six of seven alleles with the founder ST, according to the website (http://eburst.mlst.net/).

### Statistical analyses

Proportions were compared using Fisher’s exact test. The population counts and virulence scores were compared between different STs using the Mann–Whitney *U*-test in the IBM SPSS Statistics ver. 25.0 program (IBM Inc., Armonk, NY, USA). Multiple linear regression models were used to evaluate the independent contributions of ST origin (ST15, ST30, and ST45), virulence gene carriage and age of the infant on fecal population counts of *S. aureus* (SPSS Statistics ver. 25.0). Principal component analysis (PCA) was performed using the Simca-P ver. 15.0 software (Umetrics AB, Umeå, Sweden).

## Results

**Fig. 1:**
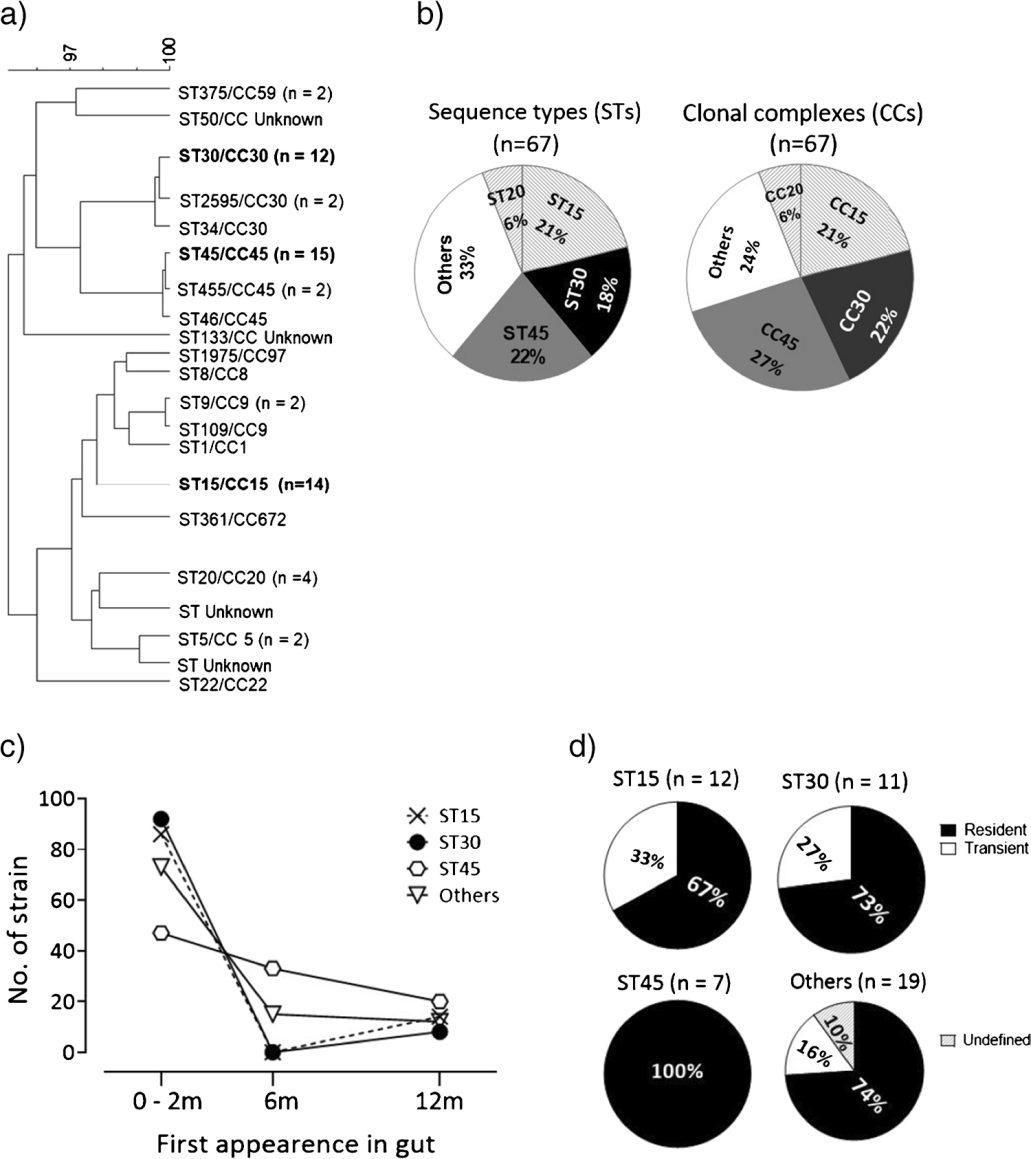
The *S. aureus* strains (*n* = 67) that colonized the guts of 49 infants during the first 12 months of life were assigned to different STs using MLST. The relationships between different STs and CCs were analyzed using eBURSTv3 (Based Upon Related Sequence Types version 3; http://eburst.mlst.net/). **a** List of all detected STs/CCs and their relatedness. **b** Distributions of the major STs (ST15, 20, 30, and 45) and the major CCs (CC15, 20, 30, and 45). **c** Time of first appearance of strains with different STs in the infants’ gut. The timing of the first appearance of each strain in the microbiota was noted, i.e., at 0–2, 6, or 12 months of age. “Others” refers to STs other than ST15, ST30, and ST45. **d** Proportions of persistent *S. aureus* strains within different STs among strains establishing in the gut microbiota of infants in the period spanning from 3 days to 2 months of age. The strains have previously been categorized as persistent (≥ 3 weeks of colonization) or transient strains (< 3 weeks of colonization) [4, 9]. “Undefined” refers to strains which could not be categorized as persistent or transient. “Others” refers to strains that did not belong to one of the major STs (ST15, ST30, and ST45)

In total, we identified 21 STs that belonged to 14 CCs among the 67 *S. aureus* strains derived from the intestinal microbiota of 49 infants (Fig. 1a). Two STs were previously unrecognized and another two STs (ST50 and ST133) could not be classified into any known CC (Fig. 1a). Two-thirds of the strains belonged to four STs, namely ST45 (22%), ST15 (21%), ST30 (18%), and ST20 (6%) (Fig. 1b), while another 15 STs comprised 1–2 strains each. The CC distribution was very similar to the ST distribution, i.e., dominated by CC45, CC30, and CC15 (Fig. 1b). Thus, all the CC15 strains (*n* = 14) belonged to a single ST (ST15), while the CC30 strains comprised three closely related STs (ST30 (*n* = 12), ST2595 (*n* = 2), and ST34 (*n* = 1)) and CC45 contained ST45 (*n* = 15), ST455 (*n* = 2), and ST46 (*n* = 1). All the following analyses were based on ST, rather than CC.

We found no relationship between colonization by any particular ST and the infant’s gender, delivery mode, or antibiotic treatment during the first year of life. Similarly, we did not detect any effect on ST distribution of exclusive breastfeeding for at least 4 months or the presence of older siblings or pets in the family (data not shown).

### ST and gut colonization pattern

The infants were followed from 3 days to 12 months of age (in all, seven sampling occasions). Figure 1c shows the age at which strains belonging to the different STs were first detected in the gut microbiota. ST45 strains were more likely than ST15 or ST30 strains to become established in the microbiota of infants who were 6 months of age or older. Thus, while 53% of the ST45 strains first appeared in the microbiota at 6 or 12 months of age, this was true for only 11% of the strains belonging to ST15 or ST30 (8/15 vs 3/26, *p* = 0.008; Fisher’s exact test) (Fig. 1c).

Strains could be persistent or transient, the former colonizing an infant for ≥ 3 weeks, the latter for < 3 weeks. As transient strains could not be identified in samples obtained at either 6 or 12 months of age, due to long sampling intervals, we included only strains establishing in the period between 3 days and 2 months of life when comparing proportions of resident and transient strains within major STs. As evident from Fig. 1d, most strains, regardless of ST, were persistent in the gut microbiota. Furthermore, all the ST45 strains that became established during this period of time were persistent and none was transient (*p* = 0.15, compared to strains belonging to ST30 or ST15; Fisher’s exact test).

We next compared the duration of colonization of persistent strains of different STs. The average duration of colonization of the persistent ST45 strains (*n* = 9) was ≥ 34 weeks, as compared to ≥ 22 weeks for persistent strains of other STs (*n* = 31) (*p* = 0.04).

Furthermore, persistent strains belonging to ST45 (*n* = 9) had a significantly longer mean colonization time than the persistent strains of ST15 origin (*n* = 9) (34 vs 19 weeks; *p* = 0.04), and they also tended to colonize longer than the persistent strains of ST30 origin (*n* = 8) (34 vs 23 weeks; *p* = 0.11).

### ST origin and fecal population counts in colonized infants

The fecal population counts of each *S. aureus* strain in each stool sample were previously determined [4]. The average fecal population counts in colonized infants decreased from roughly 10^6.8^ CFU/g in 1-week-old infants to 10^4^ CFU/g in 12-month-old infants [4], suggestively due to increased competition in the gut microbiota.

**Fig. 2:**
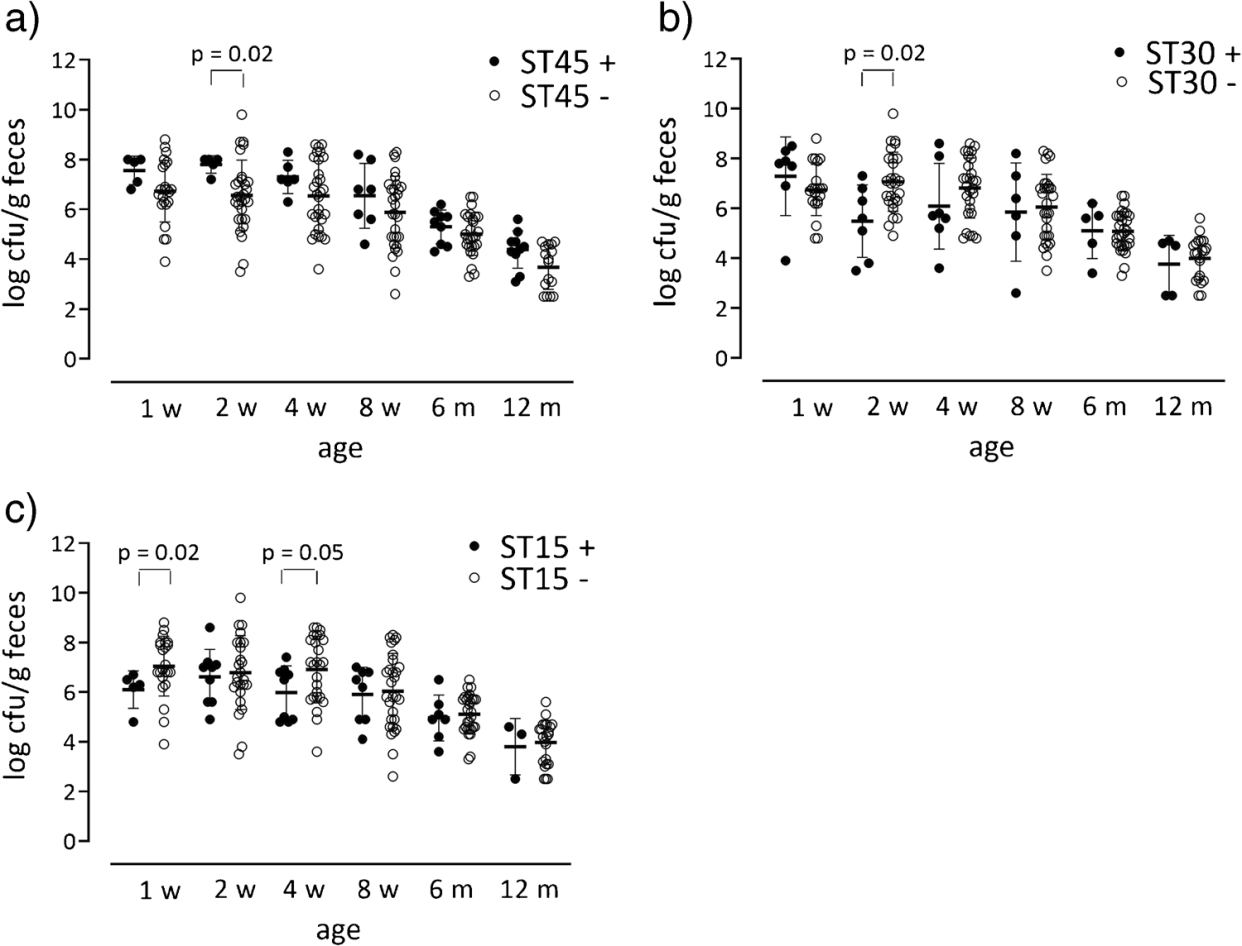
Stool population counts of 67 *S. aureus* strains of different STs that colonized the intestines of 49 Swedish infants during the first year of life. The figure depicts the population levels at 1, 2, 4, and 8 weeks of age, as well as at 6 and 12 months of age. The population counts of each major ST, i.e., ST45, ST30, and ST15, are compared to the populations of strains not belonging to that ST (**a**–**c**, respectively) using the Mann–Whitney *U*-test

Figure 2 shows the fecal population counts of strains belonging to ST15, ST30, or ST45 at different time-points, in comparison to all other strains present at the same age in the feces of these infants. Strains belonging to ST45 displayed higher population counts than those with other STs at several time-points, although the difference was statistically significant only at 2 weeks of age (*p* = 0.02) (Fig. 2a). In contrast, strains belonging to ST30 had lower population counts than other strains at 2 weeks of age (*p* = 0.02) (Fig. 2b), while ST15 strains had significantly lower population counts than other strains at 1 week (*p* = 0.02) and 4 weeks (*p* = 0.04) of age (Fig. 2c).

Multiple linear regression was used to model *S. aureus* fecal population levels as a function of age (in weeks) and ST origin. As shown in Table 1, increasing age was significantly associated with decreasing *S. aureus* population counts in fecal samples during the first year of life (Table 1). Independent of age, ST45 origin was associated with significantly higher fecal population counts compared with the other ST types. In contrast, both ST15 and ST30 tended to be associated with low fecal population levels (Table 1).

**Table 1:** Linear regression model assessing the contribution of the infant’s age and ST origin of *S. aureus* strains on its population counts in the infant gut

Variables	Regression coefficient *B*	Standard error of *B*	*p*-value
Constant	6.856	0.154	0.000
Age (weeks)	− 0.061	0.005	**0.000**
ST45	0.527	0.233	**0.025**
ST30	− 0.329	0.233	0.160
ST15	− 0.371	0.230	0.108

*S. aureus* strains isolated from feces of infants from 1 week to 12 months of age were assigned to different STs using MLST. Multiple linear regression was used to evaluate the independent contributions of age at which fecal samples were collected and ST origin of the *S. aureus* strains on the population counts of *S. aureus* strains in fecal samples. The STs included in the analysis were ST15, ST30, and ST45, since these STs showed the highest prevalence in the gut microflora of the infants studied. Statistically significant results are indicated in bold

### ST, virulence gene profiles, and fecal population counts

**Fig. 3:**
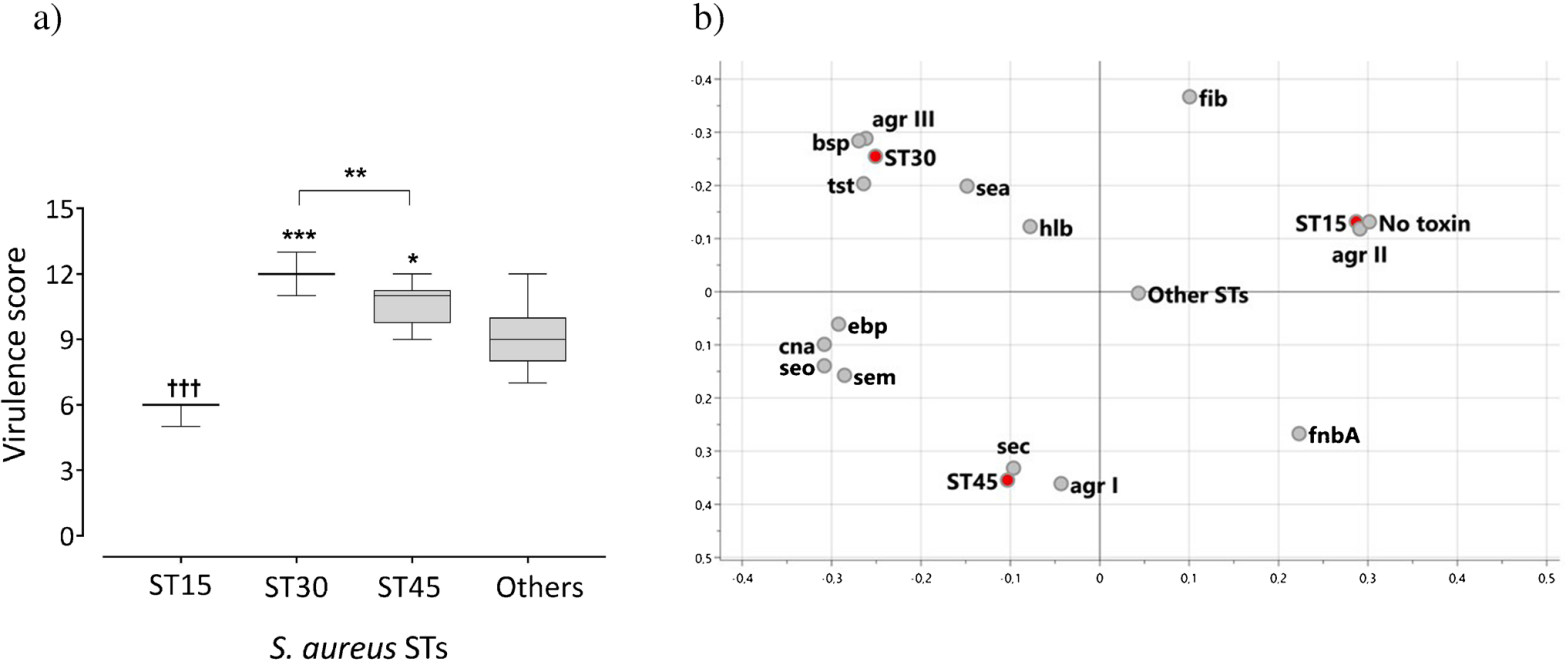
**a** Virulence scores for the major STs. A virulence score was calculated for each strain by summing the numbers of virulence genes (Table 2) possessed by that strain. The mean virulence score (± SD) was calculated for each ST. Virulence scores were compared between strains of each ST and strains of all other STs, and between the ST30 and ST45 strains using the Mann–Whitney *U*-test. ***p = 0.0001, *p = 0.02 (higher than all other strains in the strain collection); †††*p* ≤ 0.0001 (lower than all other strains in the strain collection). “Others” refers to strains that do not belong to one of the major STs, ST15, 30, and 45. Virulence scores were compared separately between the ST30 and ST45 strains. **b** Principal component analysis showing *S. aureus* virulence gene carriage in relation to ST origin. Virulence genes encoding toxins and adhesins and each of four *agr* alleles are included. “No superantigen” denotes the absence of all screened superantigen toxin genes

Different STs are associated with different *agr* alleles and different sets of virulence genes [14, 20]. Previously, we analyzed the current *S. aureus* strain collection for carriage of 30 virulence genes, including those for toxins and adhesins [9]. Here, we calculated the average number of virulence genes for each ST, to generate a “virulence score” [23, 24]. As shown in Fig. 3a, the ST30 strains had the highest virulence score, and ST15 strains the lowest score (*p* = 0.0001 in both cases, compared to all other strains). The ST45 strains also had a higher average virulence score than the remainder of the strains (*p* = 0.02), while the ST30 strains had a higher mean virulence score than the ST45 strains (*p* = 0.001) (Fig. 3a).

**Table 2:** Virulence factor-encoding genes detected in gut colonizing *S. aureus* strains belonging to different STs

Virulence traits	Gene	Carriage rate (%)			
		ST15	ST30	ST45	Others
		(*n* = 14)	(*n* = 12)	(*n* = 15)	(*n* = 26)
agr alleles					
*agr I*		0^††^	0^†^	100**	54
*agr II*		100**	0	0	27
*agr III*		0	100**	0	15
*agr IV*		0	0	0	4
Superantigen					
Enterotoxin A	*sea*	0	67**	0	15
Enterotoxin C	*sec*	0	0	86**	0
Enterotoxin L	*sell*	0	0	86**	0
Enterotoxin M	*selm*	0^††^	100	100*	65
Enterotoxin O	*selo*	0^††^	100	100	73
Toxic shock syndrome TSST-1	*tst*	0	92**	14	4
No superantigens		100^††^	0	0	4
Others					
Beta-hemolysin	*hlb*	0	33	0	11
Adhesins					
Bone sialoprotein binding protein	*bsp*	0	100**	0	11
Collagen binding protein	*cna*	0^††^	100**	100**	27
Elastin binding protein	*ebp*	0^††^	100	93	61
Fibrinogen binding protein	*fib*	100	100	7^††^	85
Fibronectin binding protein A	*fnbA*	93	17^††^	100	85
Fibronectin binding protein B	*fnbB*	100	92	100	100
Clumping factor A	*clfA*	100	100	93	100
Clumping factor B	*clfB*	100	100	93	100

*S. aureus* strains from the gut microbiota of infants were assigned to different STs using MLST. The virulence traits of individual strains were previously determined [9]. Here, the prevalence of each virulence marker is compared between strains that belong to a particular ST and all the other strains. “Others” refers to strains that do not belong to one of the major STs (ST15, ST30, and ST45). The following toxin genes were identified in ≤ 6% of the strains: *seb* (SEB), *sed* (SED), *seh* (SEH), *eta* (exfoliative toxins A), *selk* (SElK), *selp* (SElP), *ser* (SER), and *selq* (SElQ). No strain carried the toxin genes for *etb* and *etd* (exfoliative toxins B and D), *pvl* (Panton-Valentine leukocidin), or *lukM* (leukotoxin M). The adhesin genes *lbp* (encoding laminin-binding protein) were carried by 100% of the strains. “No superantigen” denotes the absence of all screened superantigen toxin genes

Proportions were compared using Fisher’s exact test and the *p*-values were corrected by the Bonferroni method for multiple comparisons. **p ≤ 0.003 (higher than all other strains in the same collection); ^††^*p* ≤ 0.003, and ^†^*p* ≤ 0.03 (lower than all other strains in the same collection)

Principal component analysis was used to illustrate the virulence gene patterns of ST15, ST30, and ST45. As shown in Fig. 3b, the ST45 strains belonged to *agr I* and carried the toxin-encoding genes *sell* and *sec*. The *sec* and *sell* genes were found exclusively in ST45 strains (Table 2) and always appeared together (data not shown). ST30 associated with *agr* III, the toxin genes *tst* and *sea*, and the adhesin gene *bsp*, as previously described [14, 20]. In addition, both ST30 and ST45 strains shared the *ebp* and *cna* adhesin genes and the superantigen genes *selm* and *selo* (encoded by the enterotoxin gene cluster) (Fig. 3b). In fact, all the ST45 and ST30 strains displayed this virulence gene pattern (Table 2). Interestingly, ST15 strains lacked all the screened enterotoxin genes (“No toxin,” Fig. 3b and Table 2). Furthermore, the ST15 strains lacked the hemolysin-encoding *hlb* gene, as well as the genes for collagen-binding and elastin-binding proteins. However, all of these strains carried the *fib* gene, and this was also true for the ST30 strains (Table 2).

Next, we investigated the influence of genetic backbone and virulence gene profile on fecal population counts using several multiple regression models. In these analyses, we included as variables the virulence genes that were previously found to be associated with *S. aureus* population counts [9], alongside STs and age of the infant. First, we investigated the impacts of ST45 and the virulence genes *selo* and *cna* on fecal *S. aureus* population counts in colonized infants. As shown in Table 3, ST45 origin was a strong determinant of high fecal population counts independent of carriage of these virulence genes (Model 1).

**Table 3:** Multiple regression models to assess the contribution of ST45, ST30, or ST15 and selected virulence genes to the population counts of *S. aureus* strains in the gut microbiota of infants

Variables	Regression coefficient *B*	Standard error of *B*	*p*-value
Model 1 (ST45)			
Constant	6.561	0.163	0.000
Age (weeks)	− 0.061	0.005	**0.000**
ST45	0.663	0.247	**0.008**
* selo*	0.234	0.229	0.308
* cna*	− 0.082	0.233	0.724
Model 2 (ST30)			
Constant	7.467	0.191	0.000
Age (weeks)	− 0.061	0.005	**0.000**
ST30	0.407	0.284	0.154
* fib*	− 0.780	0.203	**0.000**
* sea*	− 0.805	0.293	**0.007**
Model 3 (ST15)			
Constant	7.509	0.193	0.000
Age (weeks)	− 0.068	0.005	**0.000**
ST15	0.694	0.546	0.206
* fib*	− 0.791	0.205	**0.000**
* No toxin*	− 0.909	0.529	0.087

Multiple regression models to investigate the independent contributions of ST origin and their associated virulence genes to the *S. aureus* fecal population counts. The virulence genes were those previously described as essential factors regarding *S. aureus* population counts [9]. Model 1 reveals how ST45, which is associated with higher population counts (Table 1), the virulence genes *sem* and *cna*, and the age at which the sample was collected, affects the fecal population counts in colonized infants. ST15 and ST30 are associated with lower-than-average fecal *S. aureus* population counts (Table 1). Model 2 analyzes the effects of ST30 origin, the superantigen gene *sea* and the adhesin *fib* gene, and age on the *S. aureus* fecal population counts. Model 3 analyzes the impacts of ST15, carriage of the *fib* gene, lack of the screened toxin genes, and age on the fecal *S. aureus* population counts. The lack of all screened superantigen genes also seems to contribute to low population counts, albeit not significantly

ST15 and ST30 were found to be associated with lower than average fecal population counts (Table 1). In Model 2, we analyzed the contributions of ST30 origin, *sea* and *fib*, previously found to be associated with low population counts [9] and enriched in ST30 strains (Table 2). As seen in Table 3 (Model 2), both the *sea* and the *fib* gene contributed strongly to low *S. aureus* population counts, whereas the ST30 backbone tended to be associated with higher population counts. Lastly, the low population counts of ST15 strains were strongly linked to carriage of the *fib* gene. The lack of any known superantigen-encoding genes also seemed to contribute to the low population counts, albeit not significantly (Model 3).

## Discussion

In the current study, we show that ST15, ST30, and ST45 together account for 61% of the strains colonizing the gut microbiota of infants in a Swedish birth-cohort at 0–12 months of age. ST30 and ST45 have previously been identified as the most common STs in various *S. aureus* strain collections of human origin, representing both invasive and nasal commensal isolates from different geographic regions [16, 17].

In the present study, ST45 was the most common ST, representing 22% of the strains. Together with the closely related STs, i.e., ST455 and ST46, they form the CC45, which comprised 27% of the gut strains. We found no significant associations between ST origin and lifestyle factors, such as gender, delivery mode, antibiotic treatment, exclusive breastfeeding for at least 4 months, having older siblings, and pet exposure. However, our examined cohort is relatively small and not powered to investigate potential connections between ST origin and different lifestyle factors, clinical histories, etc.

We uncovered several pieces of evidence pointing to a prominent capacity of ST45 strains to colonize the gut of infants. First, the ST45 strains reached higher population counts than strains belonging to other STs. All of the ST45 strains carried the *selm*/*selo* genes encoding the superantigens SElM and SElO, as well as the adhesin gene *cna*, and all of these genes have been previously linked to higher fecal population counts in the infant microbiota [9]. However, in a multivariate analysis, none of these traits exerted an independent positive effect on the fecal population levels, which were instead linked to ST45 itself. Therefore, yet-undefined factors in ST45 appear to be responsible for the ability of this ST to expand in the gut microbiota in competition with other resident bacteria. The ST represents the genetic backbone of a strain, which could make it more or less suitable for colonization of a particular ecologic niche. In analogy, in reference to a “classical” gut colonizer, *E. coli* strains belonging to the B2 phylogenetic group have a superior capacity to colonize the infant gut, which only partly depends upon virulence/colonization factors enriched in B2 strains [25].

A second sign of a specific capacity of ST45 strains to colonize the infant gut is that none of them was transient in the microbiota, defined as persisting for less than 3 weeks. Furthermore, among the persistent strains, defined as those residing for at least 3 weeks in the microbiota, ST45 strains dwelled for a longer time in the microbiota than persistent strains belonging to other STs (34 weeks vs 22 weeks, *p* = 0.04). It should be noted, however, that regardless of ST origin, a clear majority of all strains that colonized the studied infants between 3 days and 2 months of age became established as persistent colonizers in the infant gut. This may be attributable to a low level of resistance to colonization offered by the microbiota of young infants. Furthermore, the adhesin gene *fnbB* encoding fibronectin-binding protein was carried by almost all the strains examined here; in another cohort, the carriage of this gene correlated positively with the capacity of *S. aureus* to persist in the gut microbiota of infants [5].

We have no information regarding the sources of the STs colonizing the gut of the infants studied here, since no *S. aureus* strains were obtained from individuals in contact with the infants. However, in a previous study of other infants in the ALLERGYFLORA cohort, we showed that approximately half of the *S. aureus* strains colonizing the infant gut at 0–2 months of age were derived from the skin microbiota of one or both of the parents, while half of the strains could not be tracked back to the parents [6]. We had no information regarding the distribution of different STs among these strains, although their virulence gene patterns and *agr* allelic variations were characterized as part of another study [26]. We checked these strains for the combinations of *agr* allele and virulence genes characterizing certain STs (see Table 2); 42% of the strains that were shared between infants and parent (s) showed combined characteristics resembling ST45 strains, while 25% resembled ST30 strains. Among the strains from the infants that could not be tracked to a parent or parents, only 21% shared characteristics with ST45 (*p* = 0.29), while 36% resembled ST30 strains. These observations may indicate that many of the ST45 strains in the present study originated from the parents of the infants. Another likely source of colonization is elder siblings, whom seem to chiefly contribute to infant colonization at 2–6 months of age [27].

It is notable that the majority (53%) of ST45 strains first appeared in the microbiota at 6 or 12 months of age, while this was true of only 11% of ST30 or ST15 strains (*p* = 0.008). The capacity of ST45 strains to establish themselves in the infant microbiota not only during the first period of life, when the complexity of the microbiota is limited and therefore offers little colonization resistance, but also in the second half of the first year, may further indicate their suitability as gut colonizers.

To speculate, the parents, and later elder siblings, may be a major source of ST45 strains during infancy, while ST30 and ST15 strains may be acquired more frequently in the hospital soon after birth. In that case, they will not be available once the mother returns home with her infant. This could, of course, be part of the explanation for the late acquisition of ST45, but not ST30 or ST15 strains.

ST30 and ST15 were, together with ST45, the dominant STs in the gut microbiota of the investigated infants. ST30 is one of the most-common STs, especially among isolates recovered from patients with invasive diseases [18]. In a small study from Spain, CC30 (including ST30) was recovered from 6/15 adult individuals harboring *S. aureus* in the gut [28]. In our study, the ST30 strains carried the highest number of virulence genes, and, more often than strains of other STs, they carried the superantigen genes *sea* and *tst* and the adhesin genes *bsp* and *cna*, which is in agreement with previous findings [14]. However, we found that *cna* was not unique to ST30, as it was also found in 100% of the ST45 strains. Interestingly, we found that the ST30 strains had comparatively low fecal population counts. Multiple regression indicated that this was not due to the ST30 backbone, but rather to their carriage of genes for fibrinogen-binding protein (*fib*) and for enterotoxin A (*sea*).

Our findings in another birth-cohort indicated a comparatively poor capacity of ST30 strains to colonize and persist in the infant gut microbiota. Strains that could colonize the nose, but not the gut, and strains that were only transient in the gut microbiota belonged to the *agr* III group and carried *tst*, *sea*, and *bsp* [5], which are the traits that characterize ST30 strains [14, 20]. Thus, although we did not determine the ST in the previous study, the virulence profile and distribution of particular *agr* alleles indicate that these poor colonizers of the gut were ST30 strains. In the same study, a high percentage of both nasal and gut colonizing strains showed virulence and *agr* characteristics resembling ST45 strains [5].

We have limited information regarding the ST15 lineage. It is not often listed among the major STs found in humans. It was identified in 2/15 healthy *S. aureus* gut carriers in the Spanish study [28], and it was one of the commonest STs among nasal *S. aureus* strains from children in Ghana [29]. A striking notion in the present study was that ST15 strains did not carry genes for any of the 13 superantigens screened for, a trait shared with only one non-ST15 strain. In our other birth cohort study [5], we found 13% of the strains to lack all the superantigens, and these strains tended to be transient colonizers both in the nose and gut.

Nasal carriage is a significant risk factor for infections, in that being a nasal carrier of *S. aureus* increases three-fold the risk of invasive *S. aureus* infection [30]. Intestinal *S. aureus* colonization has been documented as a potential risk factor for extra-intestinal *S. aureus* infection as well [3, 7]. As gut colonization by *S. aureus* is more common than nasal carriage of *S. aureus* in infants and young children, the gut-resident strains may provide an important reservoir for invasive disease in vulnerable individuals. In line with this, *S. aureus* is also a more-common cause of infant septicemia in highly developed countries [31, 32] than in low-income countries where *S. aureus* is an infrequent gut colonizer.

Data regarding the ST and virulence profiles of gut-resident *S. aureus* strains are scarce. To the best of our knowledge, this is the first report to identify and characterize sequence types of *S. aureus* strains that colonize the gut of healthy infants.

One limitation of our study is the relatively small sample size, 67 *S. aureus* strains from 49 infants. Another drawback is that the strain collection was assembled some 20 years ago. Thus, our results should be confirmed in larger and more recent strain collections. However, we believe our data are still of value, not least due to the scarcity of detailed data on gut commensal *S. aureus* strains.

Taken together, our results show that ST45, ST30, and ST15 constitute the majority of *S. aureus* strains in the infant gut microbiota, each exhibiting a unique pattern of virulence genes. Furthermore, ST45 strains have higher fecal population counts than other STs, persist longer, and can become established in somewhat older infants, while other STs mainly colonize during the first 2 months of life, a period during which the commensal microbiota offers limited resistance to bacterial colonization. Thus, ST45 strains seem to be particularly well-adapted to establishing themselves and persisting in the infant gut microbiota. This finding expands our understanding of the clinical epidemiology of commensal *S. aureus* carriage.

## Supplementary Information

Below is the link to the electronic supplementary material.

Supplementary file1 (XLSX 59 KB)

## Data Availability

The data that support the findings of this study are described in the supplementary material.
